# p38γ and p38δ Are Involved in T Lymphocyte Development

**DOI:** 10.3389/fimmu.2018.00065

**Published:** 2018-01-29

**Authors:** Ana Risco, Miguel A. Martin-Serrano, Domingo F. Barber, Ana Cuenda

**Affiliations:** ^1^Department of Immunology and Oncology, Centro Nacional de Biotecnología (CSIC), Madrid, Spain

**Keywords:** p38 mitogen-activated protein kinase, p38γ, p38δ, thymus, thymocyte development, lymph node

## Abstract

p38 mitogen-activated protein kinase (MAPK) signal transduction pathways are essential regulators of the immune response. Particularly, p38γ and p38δ regulate many immune cell functions such as cytokine production, migration, or T cell activation; however, their involvement in immune cell development is largely unknown. Here, we analysed the role of p38 MAPK isoforms p38γ and p38δ in T cell differentiation in the thymus and in lymph nodes, using mice deficient in p38γ, p38δ, or in both. We found that the T cell differentiation program in the thymus was affected at different stages in p38γ-, p38δ-, and p38γ/δ-deficient mice, and also peripheral T cell homaeostasis was compromised. Particularly, p38δ deletion affects different stages of early CD4^−^CD8^−^ double-negative thymocyte development, whereas lack of p38γ favours thymocyte positive selection from CD4^+^CD8^+^ double-positive to CD4^+^ or CD8^+^ single-positive cells. Our results identify unreported functions for p38γ and p38δ in T cells.

## Introduction

In the thymus, the development of T cell from CD4^−^CD8^−^ double-negative (DN) to CD4^+^CD8^+^ double-positive (DP) and then into CD8^+^ or CD4^+^ single-positive (SP) cells (a process known as positive selection) is regulated by multiple signalling pathways that induce cell-specific gene expression (1, 2). Failure of the appropriate signals occurring during T cell development can lead to immune disorders such as severe T cell immunodeficiency or autoimmune diseases. The mitogen-activated protein kinase (MAPK) signalling pathways, including extracellular signal-related kinase (ERK), c-Jun N-terminal kinase (JNK), and p38 MAPK, have been implicated in thymocyte differentiation (3–5). Of these three major MAPK pathways, the ERK pathway is known to be involved in T cell positive selection (6–8), since ERK1-deficient mice exhibit defects in T cell maturation (9). Results obtained with JNK1- and JNK2-deficient mice indicate that these kinases are not needed for the initial stages of T cell development (5, 10), although they are implicated in different aspects of T cell-mediated immune response, for example, in the subsequent differentiation of the activated CD4 T cells into Th1 or Th2 phenotype (3–5).

On the other hand, the p38 MAPK pathway has been suggested to participate in early thymocyte development. The mammalian p38 MAPK family is composed of four members, such as p38α, p38β, p38γ, and p38δ, encoded by distinct genes and all activated by phosphorylation mediated primarily by the MAPK kinases (MKK)3 and MKK6 (11, 12). p38 MAPK can be divided into two subsets, such as p38α/p38β and p38γ/p38δ, based on substrate specificities, protein similarity, expression patterns, and sensitivity to chemical inhibitors (11). Studies using a range of p38α inhibitors *in vitro*, dominant-negative or constitutively active transgenes, or the constitutive deletion of its activators, MKK6 and MKK3 to suppress p38 MAPK, have suggested a role for p38α in early T cell development and also in T cell function (5, 13–18). Thus, it has been shown that in mice that express dominant-negative forms of both MKK3 and MKK6 specifically in T cells, the positive selection of thymocytes is impaired (15). However, since MKK3 and MKK6 activate all p38 MAPK isoforms (12), some of the findings from experiments in which MKK3 and/or MKK6 have been overexpressed or constitutively deleted may be due to an effect on any of the four p38 MAPKs, and not necessarily on p38α specifically. Knockout mice for each p38 MAPK isoform have been generated and could be an adequate tool to elucidate the specific role of individual p38 MAPKs on T cell development. Gene targetting to delete p38α in the whole mouse yields an embryonic lethal phenotype (19–21); however, one study using p38α^−/−^Rag^−/−^ chimaeras showed normal T cell development in these mice, with no alterations in mature T cell proliferation (22). Moreover, lymphocyte development or cytokine production in response to LPS is not affected in p38β-null mice (23). A recent study using knockout mice in which p38α has been specifically deleted in T cells (24, 25) shows that mice with p38α-deficient T cells express elevated amounts of p38β, and that mice with T cells simultaneously lacking p38α and p38β displayed a decreased size and cellularity of the thymus, lymph nodes (LNs), and spleen compared with those of wild-type (WT) animals (24). However, p38α and p38β deletion did not prevented the thymic development of CD4^+^ and CD8^+^ T cells or their homing and maintenance in the LNs and spleen (24) suggesting that p38α and p38β are dispensable for the development of thymocytes and that other p38 MAPK isoforms might be regulators of this process.

Using mice deficient in p38γ, p38δ, or both, it has been shown that these kinases are crucial for the inflammation and the innate immune response, by controlling immune cell response (11, 26–29). Particularly, they have recently been shown to play important roles in regulating: cytokine production by macrophages and dendritic cells; T cell activation and neutrophil migration; and also in modulating tumourigenesis associated with inflammation (11, 28, 30, 31). Despite the implication of the p38 MAPKs in the immune response, their role in the normal physiology of haematopoietic cells is largely unknown. No anomalies have thus far been reported in T cell development in p38γ-, p38δ-, or p38γ/δ-deficient mice. There are nonetheless indications that p38γ and p38δ are implicated in the development of some immune cells. Measurement of mRNA levels in inflammatory cell lineages showed that p38α and p38δ are the dominant p38 isoforms in monocyte/macrophages, neutrophils, CD4^+^ T cells, and endothelial cells, whereas p38β and p38γ mRNAs are poorly expressed (32). A possible role for p38γ in haematopoiesis has been proposed based on the mRNA expression pattern for each p38 isoform during differentiation of primary human erythroid progenitor. p38α and p38γ are expressed in early and late stages, whereas p38δ mRNA is expressed only at terminal stages of erythroid differentiation (33). Nonetheless, the role of p38γ and p38δ in the development and function of other haematopoietic cells remains unclear.

In this study, we investigate the role of p38γ and p38δ in T lymphocyte development. We performed a comparative analysis of both, the T cell differentiation in the thymus and the distribution of T cell populations in the LNs, in WT mice, and in mice lacking p38γ, p38δ, or both and in mice expressing kinase-inactive p38γ. We found that the combined action of p38γ and p38δ is necessary for thymocyte development and peripheral T cell homeostasis.

## Materials and Methods

### Mice, Antibodies, and Reagents

Mice deficient in p38γ (p38γ^−/−^), p38δ (p38δ^−/−^), and p38γ/δ (p38γ/δ^−/−^) have been described (34). The generation of mice expressing inactive p38γ (p38γ^171A/171A^) was described in Ref. (35). All mouse strains were backcrossed onto the C57BL/6 strain for at least nine generations. Mice were housed in pathogen-free conditions. Anti-total p38α and anti-α-tubulin antibodies were from Santa Cruz, -p38β from Zymed, and -p38γ and -p38δ antibodies were produced and purified as described (36, 37). This study was carried out in accordance with the recommendations of national and EU guidelines, with the approval of the Centro Nacional de Biotecnología Animal Ethics Committee (Reference: CAM PROEX 316/15).

### Flow Cytometry Analysis

Thymus and LN cell suspensions were prepared; erythrocytes were lysed, and cells were counted. Cell samples were stained with combinations of fluorescently labelled antibodies to the cell surface markers CD3 (145-2C11), CD4 (L3T4, H129.19), CD8 (Ly-2, 53-6.7), CD44 (pgp1, IM7), CD25 (IL-2R a-chain), CD27 (LG.7F9), CD90.2 (Thy1.2, GL1) (from BD Biosciences and Biolegend) as indicated, and analysed in a FACScalibur cytometer (BD Biosciences). The profiles obtained were analysed using the FlowJo software (BD Biosciences).

### Immunoblotting

Proteins were resolved by SDS-PAGE and transferred to nitrocellulose membranes, which were blocked for 30 min in 50 mM Tris/HCl (pH 7.5), 0.15 M NaCl, 0.05% (v/v) Tween (TBST buffer) containing 10% (w/v) non-fat dry milk, then incubated in TBST buffer with 10% (w/v) non-fat dry milk and 0.5–1 mg/ml antibody (2 h at room temperature or overnight, 4°C). Horseradish peroxidase-conjugated secondary antibodies and the enhanced chemiluminescence reagent (Amersham Pharmacia Biotech), using the Odyssey infrared imaging system, were used for the detection of proteins.

### Statistical Analysis

Statistical analysis of data was performed applying the unpaired Student’s *t*-test, using the Prism software v. 4.0b (GraphPad).

## Results

### Thymocyte Development in p38γ^−/−^, p38δ^−/−^, and p38γ/δ^−/−^ Mice

**Figure 1:**
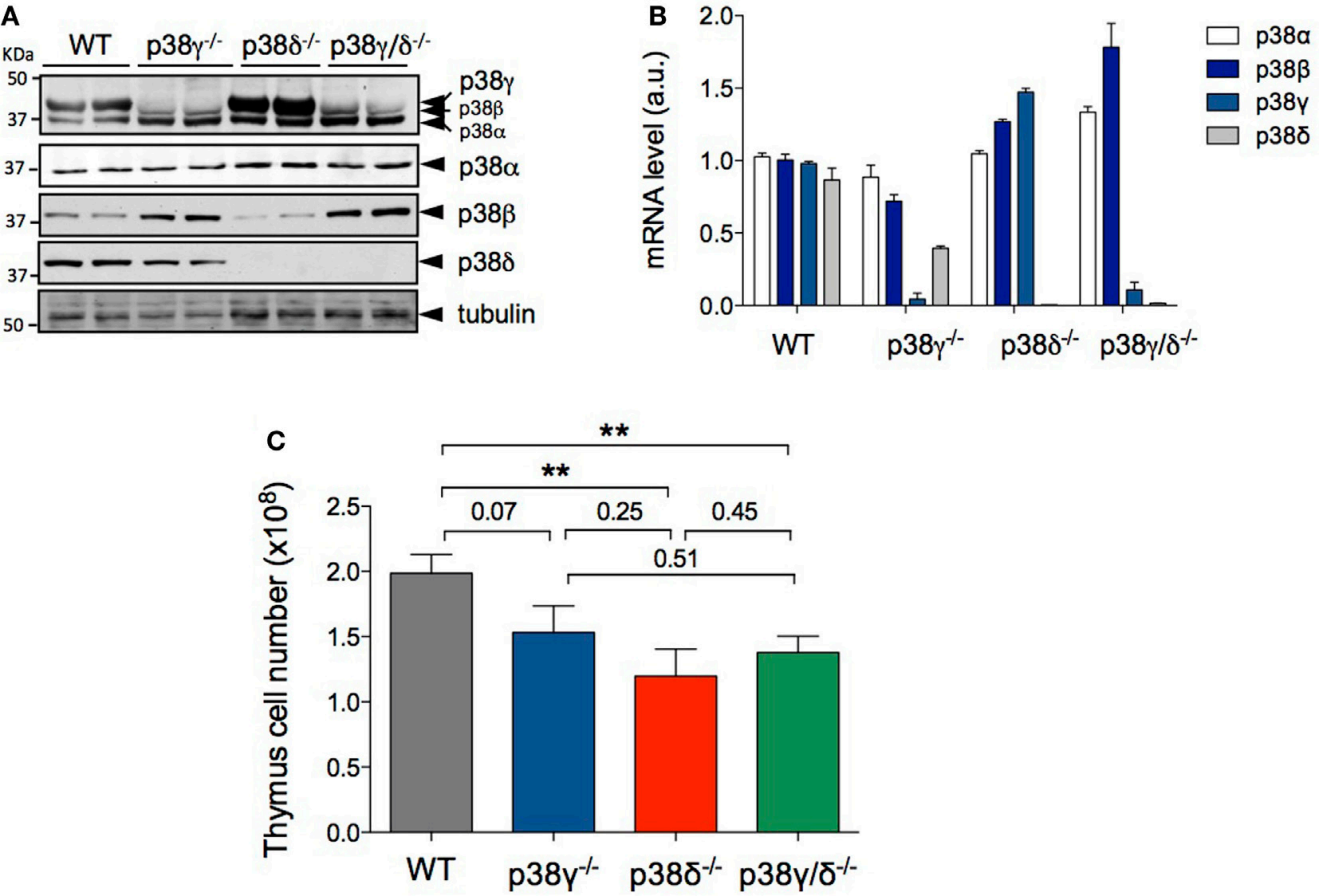
p38γ and p38δ deficiency decreases thymus cell number. **(A)** Lysates from WT, p38γ^−/−^, p38δ^−/−^, and p38γ/δ^−/−^ thymocytes were immunoblotted with anti-total p38α, -p38β, -p38γ, and -p38δ antibodies, and with anti-α-tubulin. **(B)** Expression of p38 MAPK mRNA analysed by qPCR method from WT, p38γ^−/−^, p38δ^−/−^, and p38γ/δ^−/−^ mouse thymus. Data show mean ± SD of triplicates from one representative experiment. Data were normalised to *GAPDH* mRNA. **(C)** Total cell numbers from 4-week-old (1-month) WT (*n* = 17), p38γ^−/−^ (*n* = 15), p38δ^−/−^ (*n* = 15), and p38γ/δ^−/−^ (*n* = 16) mice, determined by counting isolated cell suspensions. *p ≤ 0.05; **p ≤ 0.001.

**Figure 2:**
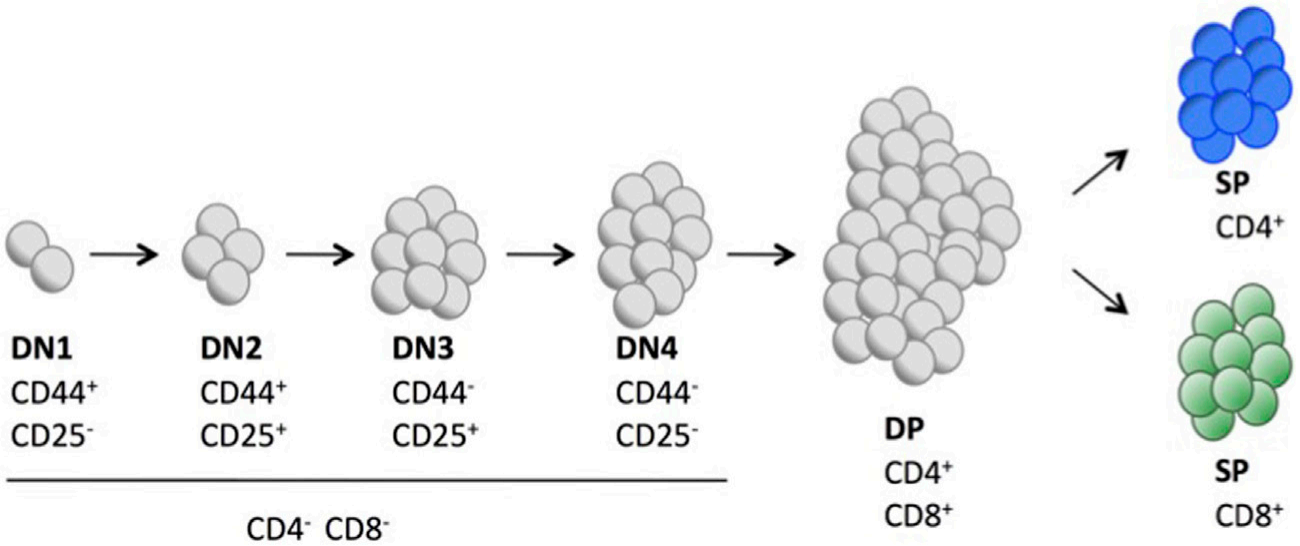
Schematic representation showing the different cell surface markers expressed in key stages of T cell development in mouse thymus.

**Figure 3:**
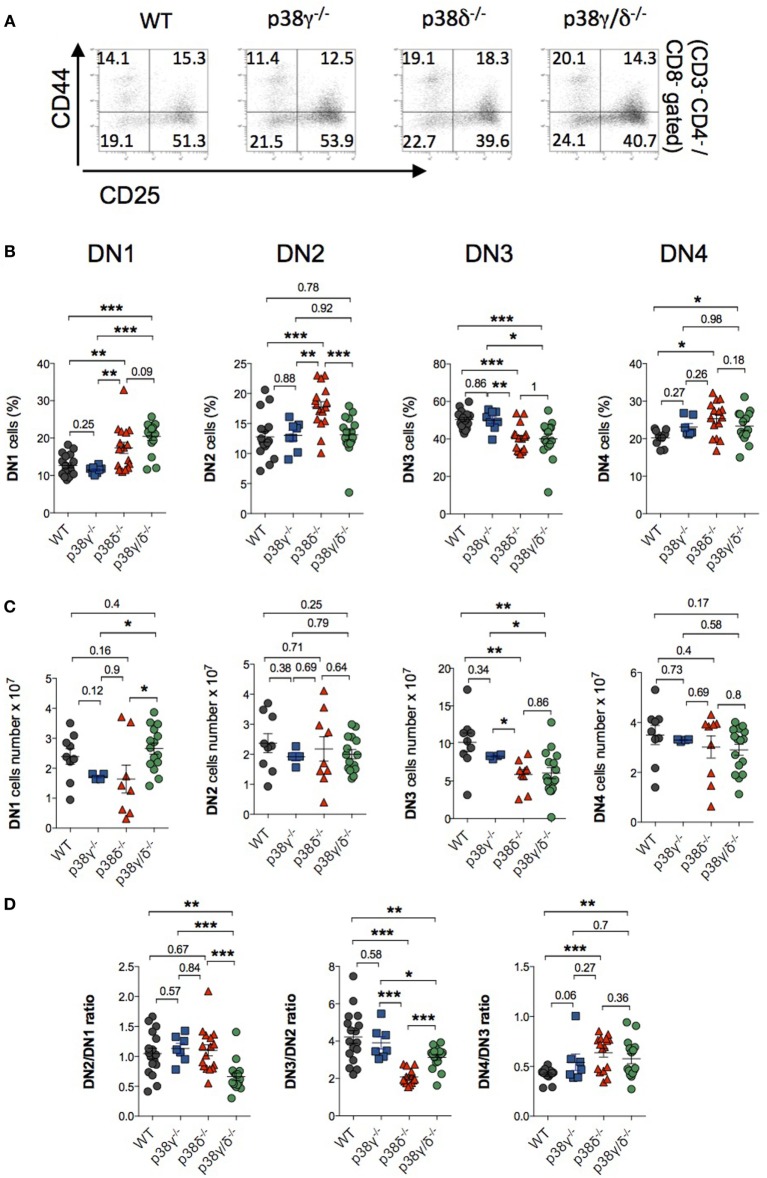
Effect of p38γ and/or p38δ deletion in the development of double-negative (DN) T cell. Thymocytes from 1-month mice were stained with anti-CD3, -CD4, -CD8, -CD44, and -CD25 antibodies and positive cells were analysed by flow cytometry. **(A)** Representative flow cytometry profiles are shown. **(B,C)** CD4^−^CD8^−^ thymocytes from 1-month mice were analysed for CD44 and CD25 expression after simultaneous staining with anti-CD44 and -CD25 antibodies. The percentages **(B)** and total numbers **(C)** of DN1, DN2, DN3, and DN4 cells were determined. Each dot represents a single mouse. *p ≤ 0.05; **p ≤ 0.001. **(D)** Ratio of DN2/DN1, DN3/DN2, and DN4/DN3 cell number. *p ≤ 0.05; **p ≤ 0.001; ***p ≤ 0.0001.

First, we examined the mRNA and protein expression of the four p38 MAPK isoforms in isolated thymocytes and observed that all p38 MAPKs were expressed in WT T cells, whereas p38γ was not expressed in p38γ^−/−^ and p38γ/δ^−/−^ T cells, and p38δ was not expressed in p38δ^−/−^ and p38γ/δ^−/−^ T cells (Figures 1A,B). Analysis of the total number of thymocyte showed decreased thymic cellularity in p38γ^−/−^, p38δ^−/−^, and p38γ/δ^−/−^ mice compared with WT mice (Figure 1C). On average, the reduction of total thymocyte number ranged from 26% in p38γ^−/−^ mice to 40% in p38δ^−/−^ mice. Since a role has been proposed for the p38 MAPK pathway in the regulation of T cell development, we compared major thymus cell populations in p38γ^−/−^, p38δ^−/−^, p38γ/δ^−/−^, and WT mice by flow cytometry. Early T cell progenitors lack both CD4 and CD8 and are called DN cells. We examined the presence of the different DN T cell subpopulations by analysing CD25 and/or CD44 expression. Early precursor is CD25^−^CD44^+^ (DN1), CD25 is then upregulated CD25^+^CD44^+^ (DN2), CD44 downregulated CD25^+^CD44^−^ (DN3) and, in the DN4 stage CD25 is downregulated, CD25^−^CD44^−^ (Figure 2). When we analysed DN population frequency in early thymocyte development (Figure 3A), we observed that the lack of p38γ did not affect the frequency of any DN subpopulation compared with WT mice. However, we found a significant increase in the percentage of CD25^−^CD44^+^ (DN1), CD25^+^CD44^+^ (DN2), and CD25^−^CD44^−^ (DN4) subpopulations, together with a reduction in the CD25^+^CD44^−^ (DN3) subpopulation in p38δ^−/−^ mice. In p38γ/δ^−/−^ mice, the frequency of DN1 and DN4, but not of DN2, was higher than in WT mice, whereas DN3 subpopulation was decreased (Figure 3B). To exclude the possibility that the observed increase of DN4 frequency in the knockout mice could be due to an increase in non-T cells, we performed an internal control by labelling the cells with anti-Thy1.2. We found that in both WT and p38γ/δ^−/−^ thymus the percentage of DN4 population gated on CD3^−^CD4^−^CD8^−^ was similar to that gated on Thy1.2^+^CD3^−^CD4^−^CD8^−^. Thus, in these control experiments, the frequency of CD3^−^CD4^−^CD8^−^ DN4 population was 20.1 ± 0.9 and the frequency of Thy1.2^+^CD3^−^CD4^−^CD8^−^ DN4 population was 19.5 ± 1.3 in WT mice. Whereas in p38γ/δ^−/−^ mice, the percentage of CD3^−^CD4^−^CD8^−^ DN4 cells was 22.7 ± 1.6 and the percentage of Thy1.2^+^CD3^−^CD4^−^CD8^−^ DN4 cells was 21.5 ± 1.02. This confirms that gated CD3^−^CD4^−^CD8^−^ thymocytes are T cells. When we analysed total cell numbers in the DN population, we found no effect of p38γ and/or p38δ-deletion in DN1, DN2, and DN4 populations, whereas the DN3 subpopulation in p38δ^−/−^ and p38γ/δ^−/−^ mice was significantly reduced compared with WT and p38γ^−/−^ T cells (Figure 3C). Comparison of DN2/DN1, DN3/DN2, and DN4/DN3 ratio in p38γ^−/−^, p38δ^−/−^, and p38γ/δ^−/−^ mice versus WT mice (Figure 3D) suggested that p38δ positively control the transition from DN2 to DN3 and negatively control the transition from DN3 to DN4, whereas p38γ and p38δ together positively regulate DN1 to DN2 transition.

**Figure 4:**
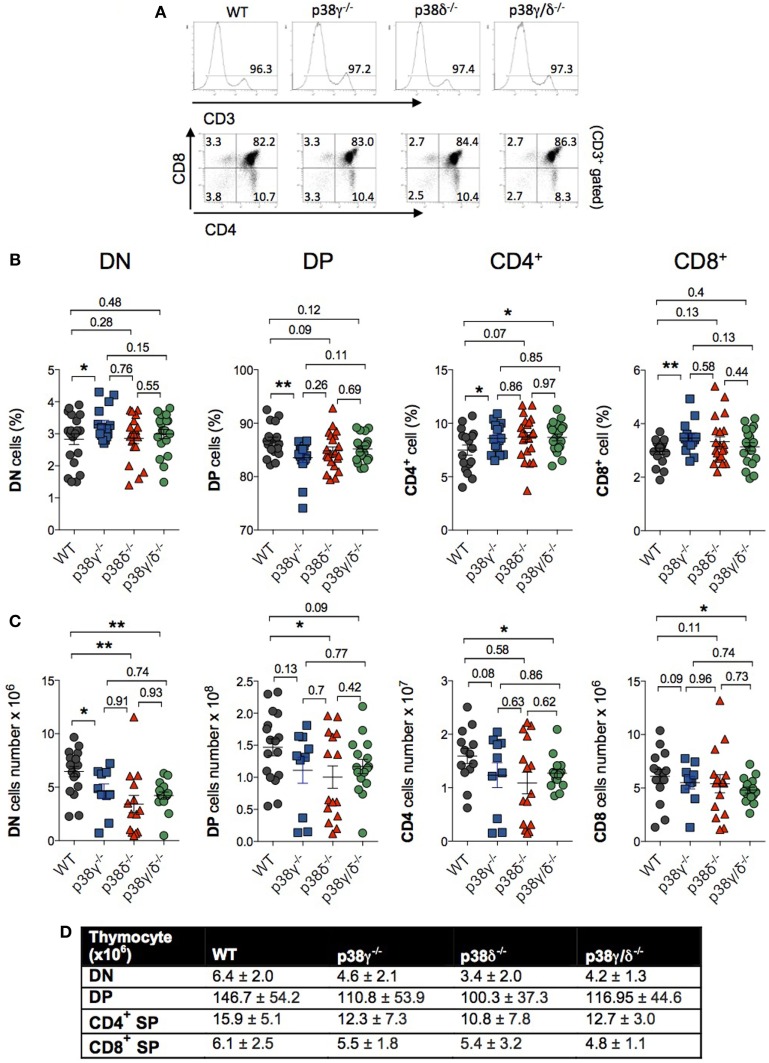
p38γ and p38δ modulate lymphoid cell development in thymus. **(A)** Thymocytes from 1-month mice were stained with anti-CD3, -CD4, and -CD8 antibodies and the percentage of positive cells was analysed by flow cytometry. Representative flow cytometry profiles are shown. **(B,C)** Thymocytes from 1-month mice were stained simultaneously with anti-CD4 and -CD8 antibodies, and the percentages **(B)** and total number **(C)** of double-negative (DN), CD4^+^, CD8^+^, and double-positive (DP) cells were analysed by flow cytometry. Each dot represents a single mouse. *p ≤ 0.05; **p ≤ 0.001. **(D)** Table showing the average cell number and the SD of thymocyte subpopulations from mice represented in panel **(C)**.

**Figure 5:**
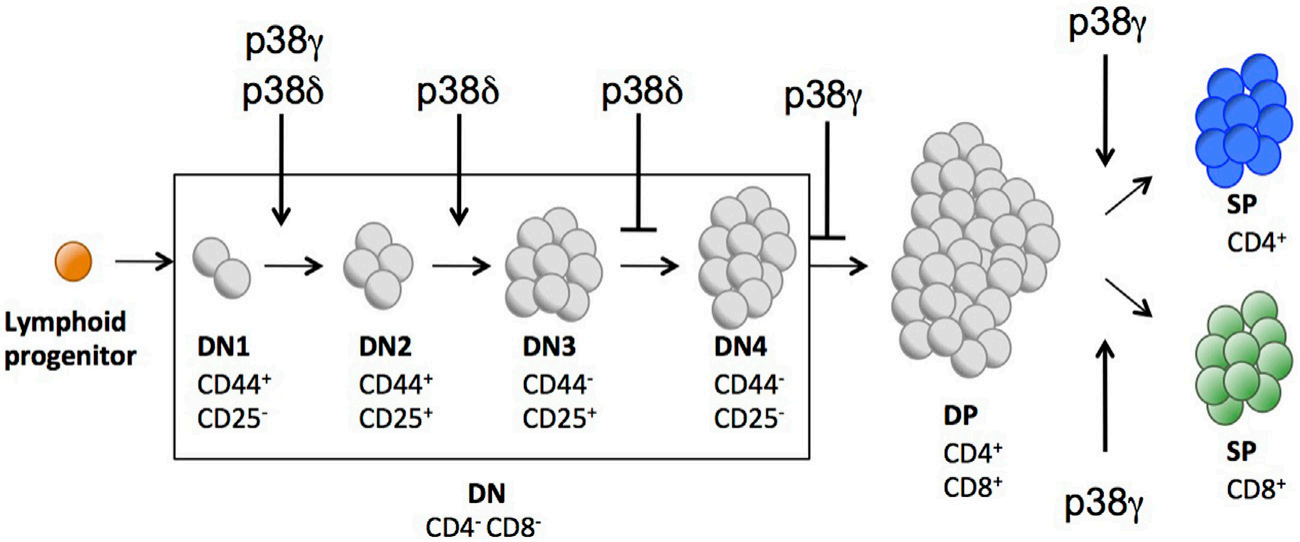
Schematic representation indicating the different stages of T cell development partially controlled by p38γ and/or p38δ.

Both CD4 and CD8 are upregulated in a next stage of T cell development to produce DP cells. Either CD4 or CD8 is then downregulated, yielding CD4^+^ or CD8^+^ SP thymocytes that move from the thymus to the periphery (Figure 2). We did not found significant differences in the percentage of DN, DP, and mature CD4^+^ and CD8^+^ SP cells between WT and p38δ^−/−^ mice. In p38γ/δ^−/−^ mice, the percentage of CD4^+^ was increased compared with WT. We also observed a moderate increase in DN and a decrease in DP thymocyte frequency in p38γ^−/−^ mice, which correlated with a proportional increase in CD4^+^ and CD8^+^ SP populations (Figures 4A,B). We found that absolute numbers of individual thymic subpopulations were slightly decreased in p38γ-, p38δ-, and p38γ/δ-deficient mice compared with WT mice (Figures 4C,D). When we analysed absolute numbers of DN, DP, CD4^+^, and CD8^+^ SP thymocytes in p38γ-, p38δ-, and p38γ/δ-deficient mice compared with WT mice, we observed a reduction from 20% (in the case of p38γ/δ^−/−^ CD4^+^ SP) to 35% (in the case of p38γ/δ^−/−^ DN) compared with WT mice (Figures 4C,D), which is consistent with the decrease in total thymocyte number (Figure 1C). All these results suggest that both p38δ and p38γ are involved in early thymocyte development by regulating different stages of differentiation in a positive or in a negative manner. Thus, p38δ and p38γ would positively control the transition from DN1 to DN2; p38δ would promote DN2 to DN3 transition and impair DN3 to DN4 transition, whereas p38γ would decrease DN to DP transition and increase positive selection (Figure 5).

**Figure 6:**
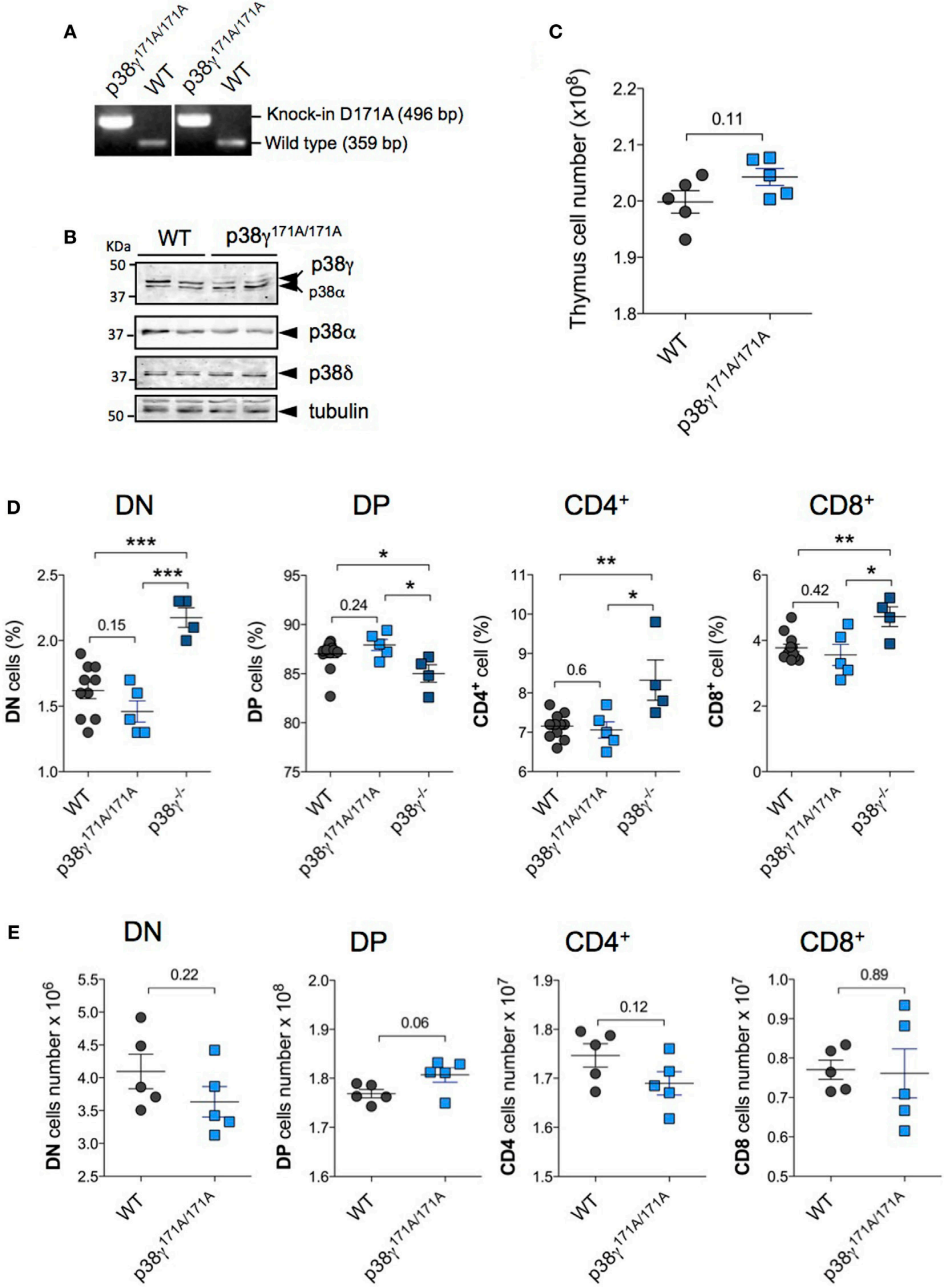
Effect of kinase-inactive p38γ (D171A) in late T cell development. **(A)** Genomic DNA purified from tail biopsy sample was used as a template for PCR as in Ref. (35). **(B)** Lysates from WT and p38γ^171A/171A^ thymocytes were immunoblotted with anti-total p38α, -p38γ, and -p38δ antibodies. **(C)** Total cell numbers from 4-week-old (1-month) WT and p38γ^171A/171A^ mice, determined by counting isolated cell suspensions. Thymocytes from 1-month WT and p38γ^171A/171A^ mice were stained simultaneously with anti-CD4 and -CD8 antibodies, and the percentages **(D)** and total number **(E)** of double-negative (DN), CD4^+^, CD8^+^, and double-positive (DP) cells were analysed by flow cytometry as in Figure 3. p38γ^−/−^ mice are included in panel **(D)** as control. Each dot represents a single mouse. *p ≤ 0.05; **p ≤ 0.001; ***p ≤ 0.0001.

Since we have previously described that p38γ can function independently of its kinase activity regulating the composition of protein complexes (35), we have checked the effect of the catalytically inactive mutant p38γ (D171A) in the transition from DN to DP and also in T cell-positive selection. p38γ^171A/171A^ mice genotype was confirmed by PCR (Figure 6A). Western blot analysis confirmed that the thymus of p38γ^171A/171A^ mouse expresses p38γ, and that the p38δ and p38α protein levels were similar to those in WT thymus (Figure 6B). Comparison of thymocyte total number from p38γ^171A/171A^ and WT mice showed no significant differences (Figure 6C). In addition, the percentage of DN, DP, and mature CD4^+^ and CD8^+^ SP cells in the thymus of p38γ^171A/171A^ were similar to WT mice, and contrary to what observed in p38γ^−/−^ mice (Figure 6D). Absolute DN, DP, CD4^+^, and CD8^+^ SP thymocyte numbers were similar between p38γ^171A/171A^ and WT mice (Figure 6E). All these results indicate that p38γ plays a role in the regulation of DN to DP transition and of T cell-positive selection independently of its canonical kinase activity.

### p38γ and p38δ Modulate αβ T Cell Development

**Figure 7:**
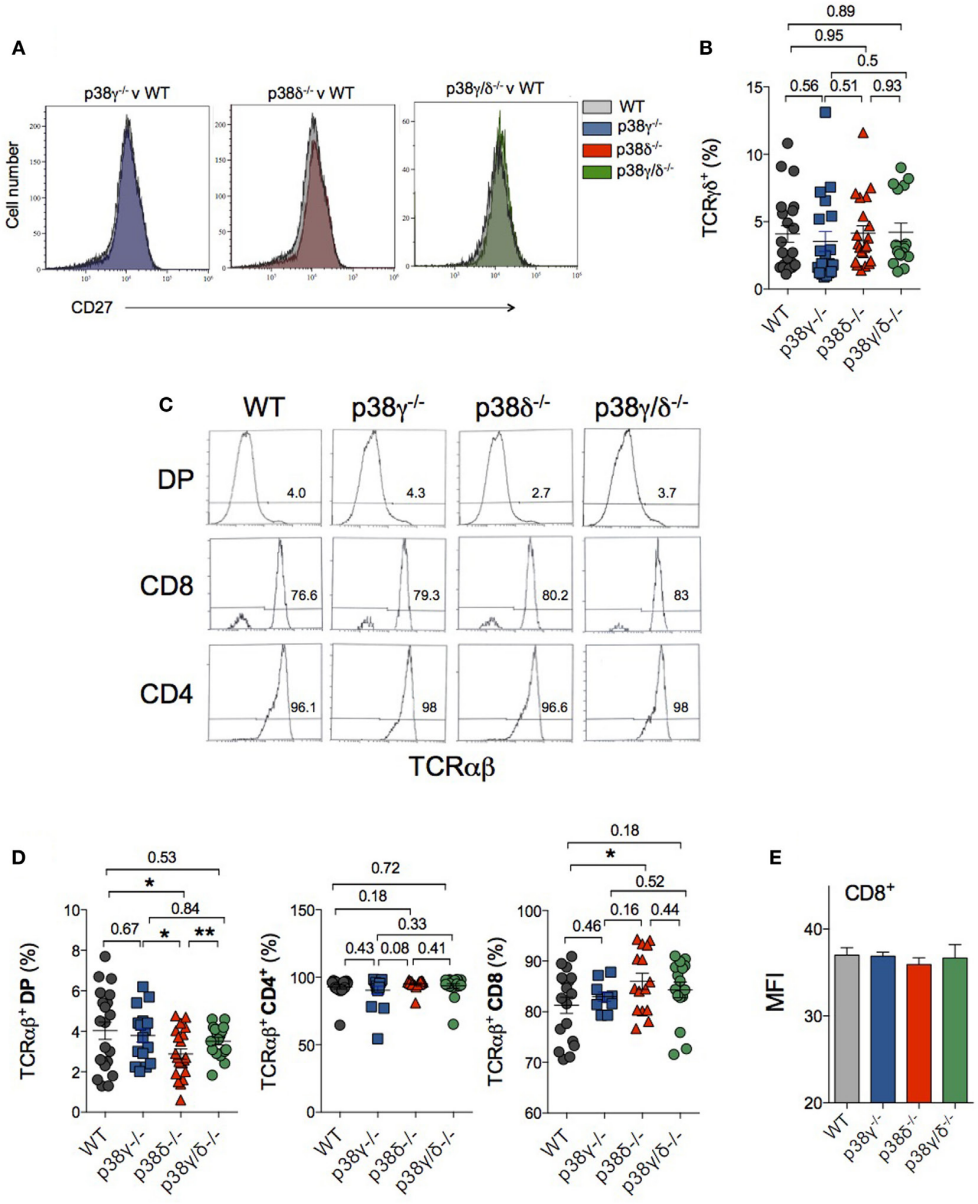
Effect of p38γ and/or p38δ deletion in the generation of γδTCR- and αβTCR-expressing T cells. Thymocytes from 1-month mice were stained with anti-CD3, -CD4, -CD8, -CD27, and -TCRγδ or -TCRαβ antibodies and cells were analysed by flow cytometry. **(A)** Analysis of CD27 expression in DN3 thymocytes. Data are representative of five different staining. **(B)** The percentages of CD3^+^, CD4^−^, CD8^−^, and -TCRγδ^+^ cells were determined. **(C)** Representative flow cytometry profiles are shown. Numbers indicate the percentage of cells falling into the respective regions. **(D)** The percentages of the different T cell populations were determined. Each dot represents a single mouse. *p ≤ 0.05; **p ≤ 0.001. **(E)** Mean fluorescence intensity (MFI) levels in αβTCR CD8^+^ cells. Data show mean ± SD from one representative experiment with five mice.

Double-negative T cells can develop to either γδ or αβ TCR-expressing cells. Successful development of T cell is dependent on signals activated by pre-TCR and TCR complex (38). Rearrangements at the *Tcrd, Tcrg*, and *Tcrb* loci are initiated at the DN2 stage, and γδ and αβ divergence is complete upon arrival at the end of DN3 stage (39). DN3 cells include pre- and post-β-selected thymocytes. We have analysed the status of β-selection of DN3 thymocytes in p38γ-, p38δ-, and p38γ/δ-deficient mice compared to WT mice checking CD27 expression by flow cytometry. The increase of CD27 expression in the DN3 stage is concomitant to cytoplasmic TCR-β expression and therefore of cells that are initiating β-selection (40). We did not observe significant differences in the expression of CD27 between genotypes (Figure 7A), indicating that p38γ and p38δ are not implicated in the process of β-selection.

To determine whether or not p38γ/p38δ contributed to commitment to the γδ and the αβ lineage, we examined TCRγδ and TCRαβ expression in thymocytes. TCRγδ expression in CD3^+^ DN, CD4^−^, and CD8^−^ thymocytes was analysed by flow cytometry using anti-γδ TCR antibodies. We did not observe significant differences in the percentage of TCRγδ^+^CD3^+^ cells between genotypes (Figure 7B).

For cells that passed along the αβ TCR pathway, DN3-stage cells first express pre-TCRα, which is encoded by a non-rearranging locus. The assembly of pre-TCR after rearrangement of the TCR β-chain triggers the transition of early DN T cells into DP. Following TCR α-chain rearrangement and the expression of the dimer TCRαβ, the binding of TCR with self-major histocompatibility complex (MHC)–peptide complex promotes DP T cell differentiation of into SP cells (1, 41). TCRαβ expression in DP, CD4^+^, and CD8^+^ thymocytes were analysed by flow cytometry using anti-αβ TCR antibodies (Figure 7C). We did not found significant differences in the percentage of TCRαβ^+^CD4^+^ cells between genotypes. However, lack of p38δ caused a slight increase in TCRαβ^+^CD8 percentage, whereas the percentage of TCRαβ^+^DP was lower in p38δ^−/−^ and p38γ/δ^−/−^ mice compared with WT and p38γ^−/−^ mice (Figure 7D). Comparison of TCRαβ mean fluorescence intensity levels in CD8^+^ cells showed not differences between genotypes (Figure 7E), indicating that the lack of p38γ/p38δ is affecting CD8^+^ expansion rather than TCR expression. These results suggest that to some extent TCRαβ expression in CD8-positive cells is dependent on p38δ signalling.

### T Cells in the Peripheral Lymphoid Organs of p38γ- and p38δ-Deficient Mice

**Figure 8:**
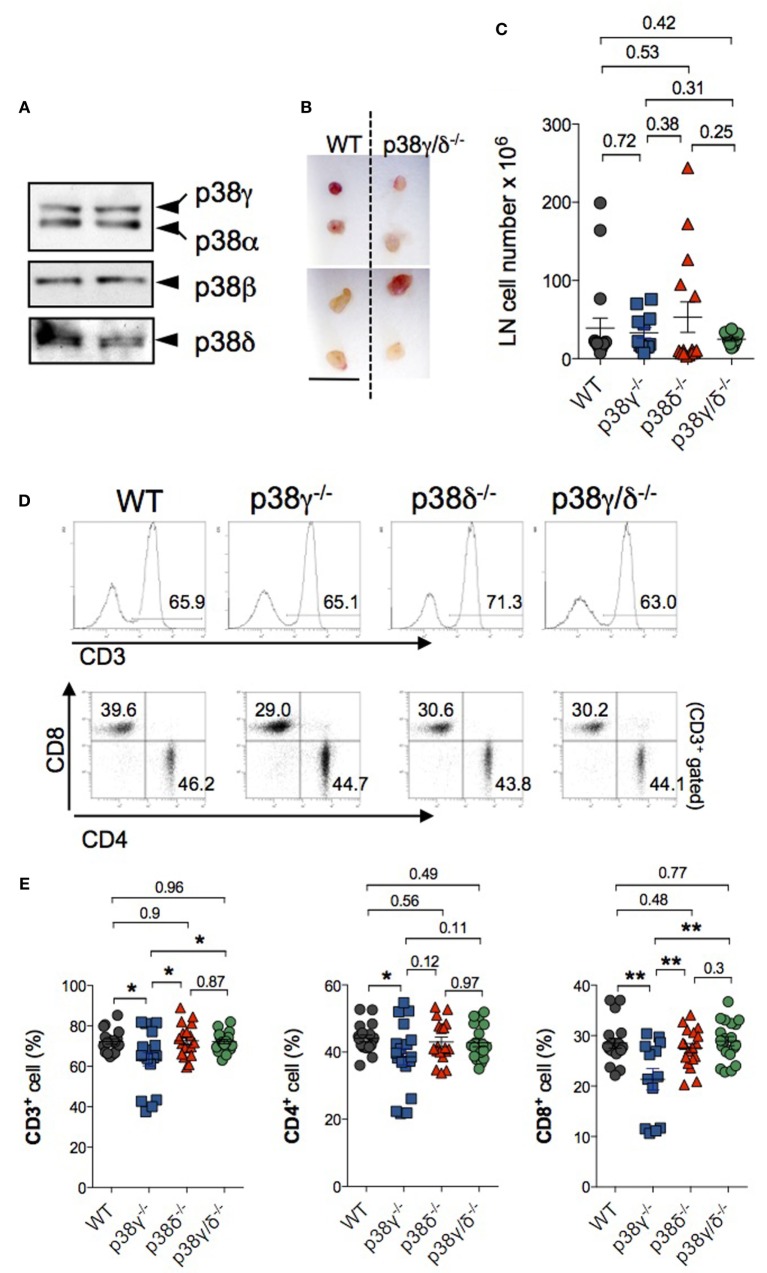

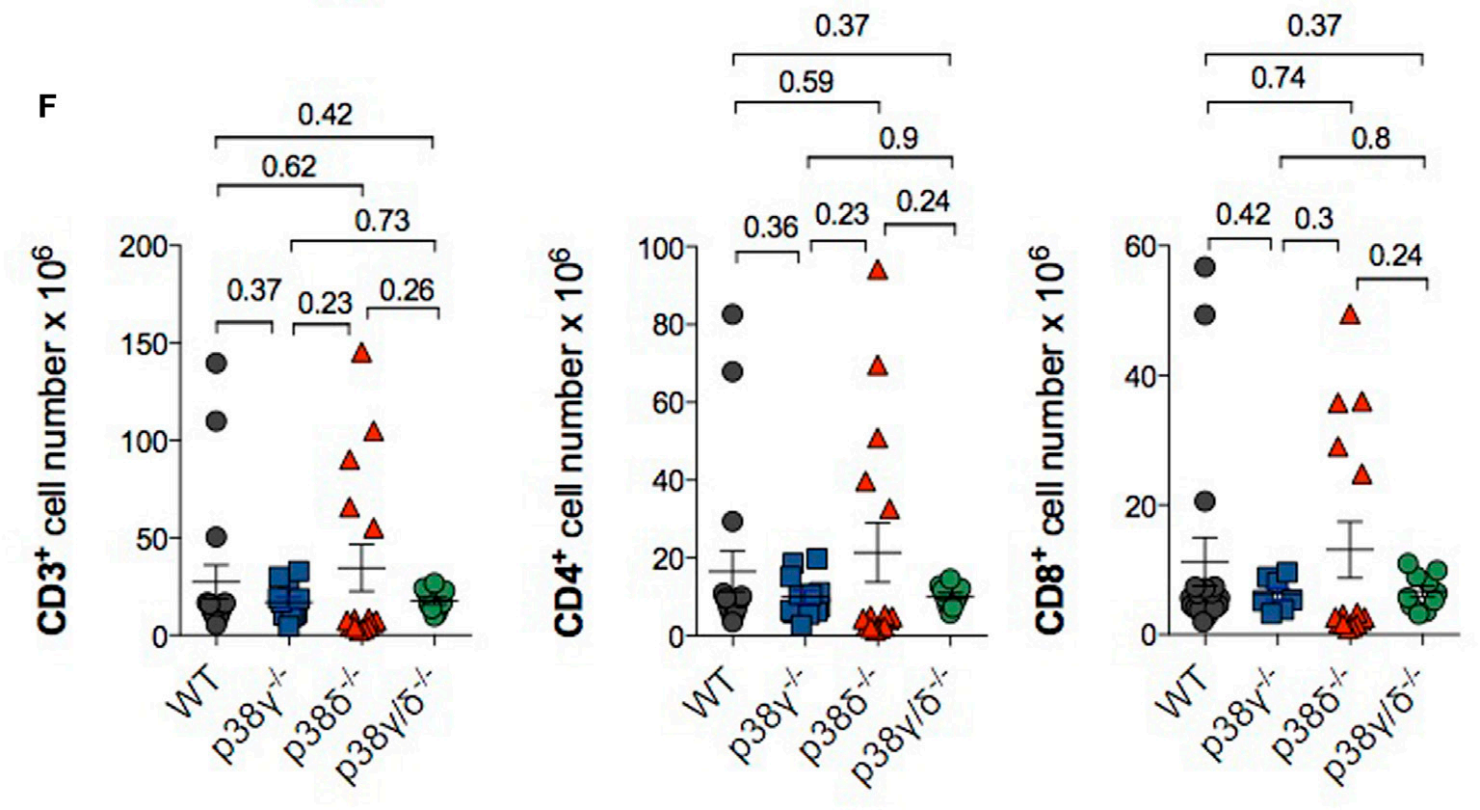
Characterisation of lymph nodes (LNs) in p38γ/δ-deficient mice. **(A)** LNs lysates from WT mice were immunoblotted with anti-total p38α, -p38β, -p38γ, and -p38δ antibodies. Results from two independent samples are shown. **(B)** Representative popliteal (top) and inguinal (bottom) LN from adult WT and p38γ/δ^−/−^ mice. Bar, 1 cm. **(C)** Total cell number in draining LNs of 4-week-old (1-month) of the indicated genotypes. Each dot represents a single mouse. **p ≤ 0.001. **(D–F)** LN cells from 1-month WT, p38γ^−/−^, p38δ^−/−^, and p38γ/δ^−/−^ mice were stained with anti-CD3, -CD4, and -CD8, and the percentage of the indicated populations was examined. **(D)** Representative flow cytometry profiles and dot plots. T cells were gated as CD3^+^ cells. **(E,F)** Graphs showing total number **(F)** and percentages **(E)** of LN T cell populations. Each dot represents a single mouse. *p ≤ 0.05; **p ≤ 0.001.

We then examined whether or not p38γ and p38δ deficiency affected T cell population in secondary lymphoid organs. We analysed CD4^+^ and CD8^+^ T cell populations in LNs of WT, p38γ, and p38δ double-deficient mice (p38γ/δ^−/−^), and also of the single knockout mice p38γ^−/−^ and p38δ^−/−^ to investigate the effect of each p38 MAPK deletion. Western blot analyses showed that both p38γ and p38δ were expressed in the LNs from WT mice (Figure 8A). The sizes of p38γ/δ^−/−^ LNs were similar to those from WT mice (Figure 8B), and comparison of LN cell number from p38γ/δ^−/−^, p38γ^−/−^, p38δ^−/−^, and WT mice showed no significant difference (Figure 8C). We then analysed T cell populations by flow cytometry and found that the number of CD3^+^, and of CD4^+^ and CD8^+^ T cells was similar in all genotypes (Figure 8F); however, the percentages of CD3^+^, CD4^+^, and CD8^+^ T cells in p38γ^−/−^ LN were significantly smaller than in the rest of genotypes (Figures 8D,E). These results indicate that p38γ might play a role in DP to SP thymocyte transition and peripheral T cell homeostasis, both depending on TCR signalling. The analysis of other LN’s immune cell populations showed not major differences between genotypes (Table 1).

**Table 1:** p38γ and p38δ are not required for lymphoid and myeloid cells development in lymph node (LN).

	LN			
	Wild type	p38γ^−/−^	p38δ^−/−^	p38γ/δ^−/−^
B220^+^	30.79 ± 4.04 (*n* = 18)	32.05 ± 9.2 (*n* = 14; *p* = 0.6)	29.31 ± 8.53 (*n* = 18; *p* = 0.51)	29.01 ± 5.99 (*n* = 20; *p* = 0.3)
CD11b^+^	4.6 ± 2.3 (*n* = 17)	4.86 ± 2.75 (*n* = 13; *p* = 0.77)	3.17 ± 1.2 (*n* = 18; *p* = 0.03)	3.58 ± 1.22 (*n* = 19; *p* = 0.1)
CD11b^+^F4/80^+^	1.59 ± 1.42 (*n* = 17)	1.61 ± 1.2 (*n* = 13; *p* = 0.96)	0.97 ± 0.46 (*n* = 18; *p* = 0.09)	1.00 ± 0.47 (*n* = 19; *p* = 0.1)
CD11b^+^Gr1^+^	1.12 ± 0.6 (*n* = 17)	1.58 ± 1.23 (*n* = 13; *p* = 0.18)	0.84 ± 0.37 (*n* = 18; *p* = 0.1)	1.61 ± 1.4 (*n* = 19; *p* = 0.18)
NK^+^	0.66 ± 0.28 (*n* = 18)	0.62 ± 0.3 (*n* = 14; *p* = 0.71)	0.52 ± 0.17 (*n* = 18; *p* = 0.08)	0.97 ± 0.82 (*n* = 19; *p* = 0.13)
NKT^+^	0.4 ± 0.26 (*n* = 18)	0.2 ± 0.11 (*n* = 11; *p* = 0.03)	0.26 ± 1.12 (*n* = 18; *p* = 0.06)	0.56 ± 0.39 (*n* = 16; *p* = 0.15)

*LN cells from WT, p38γ^−/−^, p38δ^−/−^, and p38γ/δ^−/−^ mice were stained with anti-CD3, B220, -CD11b, -Gr1, -F4/80, and NK1.1 and the percentage of the indicated populations was examined by flow cytometry*.

## Discussion

Here, we used mice lacking p38γ and/or p38δ, and mice expressing a kinase-inactive p38γ mutant to analyse the effect of these p38 isoforms on the development of T lymphocytes. Studies using p38α/p38β inhibitors and the expression of dominant-negative or active forms of p38α or MKK3 and MKK6 suggested that p38α is involved in T cell development by maintaining normal CD4^−^ CD8^−^ DN thymocyte numbers and by inhibiting formation of DP cells [reviewed in Ref. (18)]. However, neither the deletion of p38α or p38β alone nor the deletion of p38α and p38β in combination leads to thymocyte development defects (22, 23, 25). These observations do not rule out a potential role for p38α and p38β signals in T cell development, as some p38 isoforms could compensate for lack of others during this process. Here, we show that both single and the combined deletion of p38γ and p38δ led to specific thymocyte development defects. Total thymocyte cellularity and the absolute numbers of DN, DP, and SP CD4^+^ and CD8^+^ thymocytes were reduced in p38γ^−/−^, p38δ^−/−^, and p38γ/δ^−/−^ mice compared with WT mice; this reduction was more evident in p38γ/δ^−/−^ mice than in the other genotypes. In early thymocyte development, there is a reduction in the percentage of the CD25^+^CD44^−^ (DN3) subpopulation within the DN population, together with an accumulation of the CD25^−^CD44^+^ (DN1), CD25^+^CD44^+^ (DN2), and CD25^−^CD44^−^ (DN4) subpopulations in p38δ^−/−^ mice. These results suggest that p38δ regulates differentiation and expansion of DN3 thymocytes, and that has either opposite or different roles in the transition from DN2 to DN3 and from DN3 to DN4. In the regulation of DN2–DN3 transition, it is possible that p38δ controls signalling pathways, such the IL7 receptor (IL7R) or the Notch pathway, that are critical for the proliferative expansion of early pro-T cells within the thymus (2). Thus, p38δ could be mediating either the secretion of Notch ligands and/or of IL7 by stromal cells in the thymus or the signalling downstream of Notch and/or IL7R in pro-T cells, or both. In the DN3–DN4 transition, p38δ might negatively modulate proliferation or survival signalling since β-selection of DN3 thymocytes in p38δ-deficient mice is not affected compared with WT, and p38 MAPKs are downstream of the TCR receptor (42). However, further studies are required to determine the exact role p38δ in early thymocyte differentiation. p38δ^−/−^ and p38γ/pδ^−/−^ mouse phenotypes resembled those observed using the dominant-negative MKK3 and MKK6 [lck-MKK3(A)/MKK6b(A)] transgenic mice (15). These mice exhibit impaired DN thymocyte development and T-cell proliferation. Accordingly, MKK6(Glu) transgenic mice, which express a constitutively active form of MKK6, exhibit an arrest in T cell development at the DN3 stage (13). Because all p38 MAPKs are specifically activated by MKK3 and MKK6 (12), but neither p38α nor p38β deletion in mice results in thymocyte development defects (22, 23, 25, 43), our observations indicate that p38γ and p38δ signalling might account for the effects observed in T cell development using transgenic MKK3 and MKK6 mice. We show that p38γ and p38δ signalling control at least partially the correct development and maintenance of the different thymocyte subsets.

Also, our data suggest that these p38 MAPKs have a role in transmitting signals from the pre-TCR during DN to DP transition and αβ TCR-mediated negative selection signals during DP to SP transition. In late thymocyte development, signals mediated by the pre-TCR complex induce CD4^−^CD8^−^ (DN) thymocyte differentiation into CD4^+^CD8^+^ (DP). After TCR α-chain rearrangement and TCRαβ expression, DP thymocyte go through both positive and negative selection after the interaction of the TCR with the corresponding MHC, which leads to the downregulation of either CD8 or CD4, and to the differentiation of DP thymocytes into CD4^+^ or CD4^+^ SP cells (1). The lack of p38γ causes a moderate increase in DN and a decrease in DP thymocyte frequency, suggesting that p38γ modulate cell survival probably by modifying TCR signal strength and affecting the threshold between positive and negative selection. Also, there is an increase in CD4^+^ and in CD8^+^ populations in the thymus of p38γ^−/−^ mice, indicating a role of this kinase in cell survival. Interestingly, the effect of p38γ in late thymocyte development is not mediated by its kinase activity since the expression of inactive mutant p38γ (D171A) reverts the effect observed in the p38γ^−/−^ mice. p38γ is the only MAPK that has a C-terminal sequence that docks directly to PDZ domains of different proteins (11, 34, 35). This allows p38γ to regulate the composition of protein complexes independently of its kinase activity in different cellular compartments (11, 34, 35). For example, p38γ binds to the scaffold protein human discs large (hDlg, also known as Dlg1 and dlgh1) modulating the integrity and composition of hDlg complexes in the nucleus and in the cytoskeleton (11, 34, 35). In T cells, hDlg is implicated in TCR-mediated actin polymerisation, and it is essential for the preservation and regulation of cell polarity, which is important for T-cell development (44). Whether or not p38γ modulates hDlg complexes, TCR affinity and T cell proliferation in late thymocyte development are being investigated.

Analysis of secondary lymphoid organs showed that lack of p38γ decreased the frequency of CD4^+^ and CD8^+^ T cells in LNs and supports the role for p38γ in mature T cell differentiation. This reduction could be due to increased apoptosis or to a defect in cell proliferation. We have previously shown in *in vitro* T cell assays that LN cells from p38γ/δ^−/−^ mice showed reduced proliferation, and interferon γ and IL-17 production, compared with WT mice, in response to anti-CD3 (30).

An important observation in this study is that in some stages of thymocyte development the combined deletion of p38γ and p38δ do not causes the same effect that the individual deletion of these kinases. For example, in p38γ/δ^−/−^ mice, the percentage of DN2 thymocytes is similar to WT, whereas in the p38δ^−/−^ mice, DN2 subpopulation is increased; in late T cell development, changes in the percentages of DN, DP, and CD8^+^ SP caused by the lack of p38γ are not observed in p38γ/δ^−/−^ mice; and combined p38γ and p38δ deletion do not have any effect on CD4^+^ and CD8^+^ T cell frequency in LN, whereas these thymocyte populations are decreased in p38γ^−/−^ mice. It is likely that compensatory mechanisms are acting in p38γ/δ^−/−^ mice to overcome the loss of p38γ and p38δ. For example, we found that the protein expression level of p38β is increased in p38γ^−/−^ and p38γ/δ^−/−^ thymocytes compared with WT cells (Figure 1A). In future, studies will be crucial to determine changes in the levels of protein expression and activation of the different p38 MAPK isoforms or other MAPKs during T cell development. Further analyses are required to establish the exact molecular mechanism(s) by which p38γ and p38δ control some stages of T cell development, and conditional knockout mice in combination with different double, triple, or quadruple knockout mice for p38α, p38β, p38γ, and p38δ should provide important information for better understanding the physiological functions of these kinases.

## Ethics Statement

This study was carried out in accordance with the recommendations of national and EU guidelines, with the approval of the Centro Nacional de Biotecnología Animal Ethics Committee (Reference: CAM PROEX 316/15).

## Author Contributions

AC, AR, MAMS and DFB designed experiments, performed experiments, and analysed the data. AC wrote the manuscript.

## Conflict of Interest Statement

The authors declare that the research was conducted in the absence of any commercial or financial relationships that could be construed as a potential conflict of interest. The reviewer MT declared a shared affiliation, though no other collaboration, with the authors to the handling editor. The reviewer HH and handling Editor declared their shared affiliation.
